# Modeling the emergent metabolic potential of soil microbiomes in Atacama landscapes

**DOI:** 10.1186/s40793-025-00749-8

**Published:** 2025-11-16

**Authors:** Constanza M. Andreani-Gerard, Natalia E. Jiménez, Ricardo Palma, Coralie Muller, Pauline Hamon-Giraud, Yann Le Cunff, Verónica Cambiazo, Mauricio González, Anne Siegel, Clémence Frioux, Alejandro Maass

**Affiliations:** 1 Sorbonne Université, CNRS, Laboratoire d’Océanographie de Villefranche (LOV), Villefranche-sur-Mer, France; 2 Center for Mathematical Modeling, University of Chile (CNRS IRL2807), Santiago, Chile; 3 Millennium Institute Center for Genome Regulation, Santiago, Chile; 4 Institute for Biological and Medical Engineering, Pontificia Universidad Católica de Chile, Santiago, Chile; 5 Department of Chemical and Bioprocess Engineering, School of Engineering, Pontificia Universidad Católica de Chile, Santiago, Chile; 6 Inria, Univ. Bordeaux, INRAE, 33400 Talence, France; 7 Univ Rennes, Inria, CNRS, IRISA, 35000 Rennes, France; 8 Bioinformatic and Gene Expression Laboratory, INTA, University of Chile, Santiago, Chile; 9 Department of Mathematical Engineering, University of Chile, Santiago, Chile

**Keywords:** Microbial communities, Metagenomics, Metabolic potential, Metabolic network, Metabolic modeling, Community-wide, Genome-resolved, Atacama Desert

## Abstract

**Background:**

Soil microbiomes harbor complex communities from which diverse ecological roles unfold, shaped by syntrophic interactions. Unraveling the mechanisms and consequences of such interactions and the underlying biochemical transformations remains challenging due to niche multidimensionality. The Atacama Desert is an extreme environment that includes unique combinations of stressful abiotic factors affecting microbial life. In particular, the Talabre Lejía transect is a natural laboratory for understanding microbiome composition, functioning, and adaptation.

**Results:**

We propose a computational framework for the simulation of the metabolic potential of microbiomes, as a proxy of how communities are prepared to respond to the environment. Through the coupling of taxonomic and functional profiling, community-wide and genome-resolved metabolic modeling, and regression analyses, we identify key metabolites and species from six contrasting soil samples across the Talabre Lejía transect. We highlight the functional redundancy of whole metagenomes, which act as a gene reservoir, from which site-specific adaptations emerge at the species level. We also link the physicochemistry from the puna and the lagoon samples to metabolic machineries that are likely crucial for sustaining microbial life in these unique environmental conditions. We further provide an abstraction of community composition and structure for each site that allowed us to describe microbiomes as resilient or sensitive to environmental shifts, through putative cooperation events.

**Conclusion:**

Our results show that the study of multi-scale metabolic potential, together with targeted modeling, contributes to elucidating the role of metabolism in the adaptation of microbial communities. Our framework was designed to handle non-model microorganisms, making it suitable for any (meta)genomic dataset that includes high-quality environmental data for enough samples.

## Background

Soil prokaryotic communities are particularly heterogeneous and complex [1]. They demonstrate specific functional responses to their environment and can modify their surroundings by passively releasing or actively secreting metabolites [2]. By sharing labor costs to adapt to environmental constraints, microbial communities exhibit self-organizing properties such as syntrophy for dealing with nutrient availability [3–5].

From an ecological perspective, synergistic interactions within microbial communities often arise from syntrophic behaviors between species [6, 7]. Several studies suggest that metabolic exchanges enable re-utilization of metabolites released into the environment, benefiting not only the producer but also neighboring populations. These molecules have been referred to as “public goods” [8] and could explain the evolution of “metabolic handoffs” [9]. Therefore, the intrinsic metabolic dependencies in cross-feeding shape community composition and stability, as microorganisms compensating for others’ gene loss affect the ability of the whole system to overcome perturbations [5, 10, 11].

In line with this, keystone species, a term originally defined in the context of food web complexity [12], correspond to members whose removal can cause a dramatic change in microbiome structure [13]. Though often unabundant and context-dependent, they play a critical role in ecosystem functioning due to their catalytic effects on metabolic hubs as they provide load points for the connectivity of the community’s network [14]. Thus, metabolism, the frontline response of organisms to environmental shifts, is a powerful analytical layer when studying community assembly and microbial interactions [15–18].

While computational approaches have been developed to unravel the taxonomic and functional architecture of soil microbiomes [19], providing mechanistic insights into how community-level properties emerge from environmental constraints and nutrient availability remains a puzzling challenge [20]. Metabolic modeling has proven useful in studies delving into population-level niches across the globe [21], inter-species interactions [22–24] and niche overlaps [25], while holding promising value for metagenomic applications [26] either looking at microbial communities as a whole [27] or analyzing its individuals through metagenome-assembled genomes (MAGs) [28].

Given that extensive microbial diversity in soils often prevents genome reconstruction for non abundant populations [29, 30], we propose to build multi-scale metabolic models, i.e., for both metagenomes and MAGs. We argue that, by capturing the combined metabolic capabilities of whole communities while highlighting single species, we can improve our understanding of the links between the environment and microbiome structure and functioning [31]. We hypothesize that the metabolism of thriving taxa reflects niche-specific strategies and that the collective metabolic repertoire, consequence of community-wide interactions, enables broader niche occupancy.

Here, we explore these questions using genome-scale metabolic models (GEMs) of soil microbiomes to infer their putative ability to produce metabolites potentially driven by the environment. To achieve our goal, we take advantage of metagenomic data and soil measurements from six sites along the Talabre-Lejía transect (TLT, $\sim$23.5$^\circ$S) in the Atacama Desert, the most arid nonpolar environment on Earth [32]. The TLT has been extensively studied for its salinity, drought, UV radiation, and extremely low availability of nutrients, uncovering key processes associated with microbial and plant adaptation [33–36]. We build on this knowledge and analyze its unique landscapes through the lens of systems biology [72–74].

## Methods

### Geographical locations and sample collection

Soil sampling, DNA extraction, and sequencing from six sites along the TLT have been previously described [37]. Briefly, bulk soil samples (100 g) were collected in triplicate at a depth of 10 cm spanning an altitudinal gradient with different vegetation covers: pre-puna (S1, 2400 to 3300 meters above sea level - m.a.s.l.), puna (S2, 3200 to 4000 m.a.s.l.), and steppe (S3 to S6, 4000 to 4500 m.a.s.l.); and stored in dry ice until arrival at the laboratory for metagenomic sequencing. Soil physicochemical measurements are presented in Table S1.

### Metagenomic sequencing

DNA extraction was performed using the NucleoSpin Food kit (Macherey-Nagel). In order to ensure sufficient yields of DNA in such challenging type of microbiomes, triplicates were pooled into one representative DNA sample per site. Sequencing was performed by MR DNA (www.mrdnalab.com, Shallowater, TX, USA) on a MiSeq platform (Illumina, San Diego, CA) with an overlapping $2\times 150$ bp configuration.

Nearly 453 million paired-end reads of 150 nucleotides in length were generated across the six sampled sites. In total, 325,603,002 of the nearly 430 million high quality reads were mapped to the assembled metagenomic contigs (76%), averaging more than 50 million reads per sample (Table S2).

### Metagenome and MAG assembly

Metagenomic reads from the six sequenced samples were trimmed using the BBTools protocols [38] to remove Illumina adapters and low quality bases. High quality reads were then used to build the metagenomic assemblies for each sample using MEGAHIT v1.2.9 [39] with the kmer preset “meta-large” recommended for soils. Statistics of the assemblies are available in Table S2. A multi-process pipeline was prepared to extract site-specific metagenome-assembled genomes (MAGs), i.e., a triple binning and consensus approach with Maxbin2 v2.2.7 [40], MetaBAT2 v2.2.15 [41] and Concoct v1.1.0 [42]. All binners were set to a minimum contig size of 2000 bp and the coverage mapping recommended by MetaBAT2. To combine and refine all outputs, the MetaWRAP pipeline [43] was used as the consensus method with parameters set to default values. Resulting bins were quality-assessed using CheckM v1.2.2 [44] and filtered to 136 MAGs (completeness >70% and contamination <10%). Taxonomic classification was performed using the GTDB release 214 with its toolkit v2.3.0 [45]. Dereplication of MAGs classified to the same species was performed following the dRep scoring metric [46] with default values for completeness, contamination, strain heterogeneity, N50, and size, and with F set to 0. The genomic dataset was reduced to 120 unique MAGs (Table S6). Relative abundance of MAGs was calculated individually using the metagenomic reads  from their site of origin, following the CoverM v0.7.0 protocol (https://github.com/wwood/CoverM).

### Taxonomic and functional profiling

Taxonomic classification of reads mapping to the metagenomic contigs was performed using the mOTUs microbial profiler v3.1 [47] with default settings (Table S3). OTU data were rarefied to the least sequenced sample using the $\texttt{rarefy\_even\_depth}$ function from the phyloseq package v1.46.0 [62] for calculating diversity within microbiomes only. Alpha diversity indices were calculated using the $\texttt{diversity}$ and $\texttt{fisher.alpha}$**fisher.alpha** functions from the vegan package v2.6 [63] in R v4.3.2 [66]. Taxonomic abundances from OTU data is summarized by phylum, class, order, family, and genus in Table S4.

Structural gene annotation was performed using Prodigal v2.6.3 [48]. Functional annotation of these genes was performed using eggNOG-mapper v2.1.6 [49] based on eggNOG orthology data release 5.0 [50] and DIAMOND v2.1.8 [51] for sequence searches. Functional categories from COG [52], PFAM [53], CAZYme [54] and KEGG [55] were extracted for analysis. Genbank files containing annotations and sequences were generated using in-house scripts (https://github.com/rpalmavejares/meta_gbk_generator) based on the SeqIO Biopython library release 1.83 [56]. Raw counts of annotated genes were normalized by the total number of entries in each functional category per sample (Table S5).

### Metabolic network reconstruction and modeling

The input to the metabolic network reconstruction was the collection of genbank files described above. Reconstruction was performed using the GeMeNet pipeline (https://gitlab.inria.fr/slimmest/gemenet) based on Pathway-tools v.25.5 [57], mpwt v0.7 [58] and Padmet v5.0.1 [59]. Reports of unique reactions per site by dataset can be found in Table S7.

Simulations of producible metabolites, were performed with Menetools v3.3.0 [59] for metagenomes and with Metage2Metabo v1.5.2 [58] for MAGs, using the commands scope and metacom respectively. Both tools require a list of available nutrients, referred to as *seeds*, which initialize the inference of other reachable compounds in the network. This step is referred to as *network expansion* and, in the genome-resolved approach, it takes into account the metabolic complementarity of MAGs, therefore informing the producibility of new metabolites from putative cross-feeding interactions. We describe as *MetaG-GEM* a metabolic model obtained from a metagenome-scale metabolic network, and as *MAG-GEM* a metabolic model resulting from a collection of MAG-scale metabolic networks. Thus, twelve systems were developed: 6 MetaG-GEMs and 6 MAG-GEMs, one of each per site.

Five conditions were designed to simulate the systems, each described as a list of seeds. The first condition is *basal medium*, comprising inorganic carbon sources (CO_2_ and HCO_3_), water, oxygen, inorganic phosphorus, nitrate, nitrite, ammonium, sulfate, sulfite, hydrogen peroxide, arsenate, molybdate, metal ions (Fe^2+^, Mg^2+^, Ni^2+^, Co^2+^ and Cu^2+^) and other coenzymes and cofactors. This basal medium is included in all four additional conditions. The *simple sugars* condition contains carbohydrates that enter glycolysis before 2-phosphoglycerate, as defined in “Group A” by Wang et al. [60] (e.g., glucose, maltose, galactose, arabinose, sorbitol and glycerol). The *complex sugars* condition includes soil organic matter such as the molecules obtained in an untargeted metabolomics effort by Swenson et al. [61] (e.g., trehalose, sucrose, rhamnose, mannitol, xylitol, linoleic acid, spermidine, coumarate and chorismate). The *all amino acids* condition includes the twenty genomically-encoded amino acids. Finally, the *non-sulfured amino acids* condition is the latter set excluding the sulfur-containing amino acids (cysteine and methionine). The conditions are summarized in Table 1 and detailed in Table S8.

**Table 1:** Summary of the five conditions constructed for simulations in terms of compounds available for initializing the network expansion

BIOAVAILABLE NUTRIENTS	basal medium	simple sugars	complex sugars	non-sulf. amino acids	all amino acids
organic carbon	0	glucose, maltose, galactose, +4	rhamnose, xylitol, spermidine, +18	ser, pro, val, thr, ile, leu, gln, lys, his, phe, arg, tyr, gly, ala, asp, asn, glt, trp	
organic nitrogen	0	0	0		
organic sulfur	0	0	0	0	cys, met
Subtotal	0	7	21	18	20

BASAL MEDIUM					
inorganic carbon			HCO_3_, CO_2_		
inorganic nitrogen			N_2_, ammonium, nitrate, nitrite		
inorganic sulfur			HS, SO_3_, S_2_O_3_, sulfate		
other inorg. chem.			H_2_O, O_2_, H_2_, H^+^, H_2_O_2_, Pi, Cl^-^, +2		
metal ions			Mg, Fe, Ni, Co, Cu, MoO_4_		
coenzymes			NAD, FAD, CoA, thiamine, +14		
Subtotal	43	43	43	43	43
Total	43	50	64	61	63

In total, 60 simulations were performed, the 12 systems with the 5 conditions, generating lists of producible metabolites for each.The log files containing the output lists were used to construct a binary matrix as each simulation was summarized in a binary vector describing the producibility (1) or non-producibility (0) of all metabolites. Identical presence/absence vectors were collapsed into *metabolite groups*, i.e., unique binary vectors across simulations (Table S9). Metabolites predicted in MAG-GEMs that were never predicted in MetaG-GEMs were removed to eliminate the effect of gap-filled reactions during the network reconstruction step (n = 82).

### Ordination and regression analyses

To determine the taxonomic ranks and functional categories that contributed most to the differences in abundance between the six soil microbiomes, a similarity percentage analysis (SIMPER) was performed using the Bray–Curtis dissimilarity upon the Hellinger-transformed data in PAST v4.03 [64].

Principal Coordinate Analyses (PCoA) were performed with stats::cmdscale using the Bray–Curtis dissimilarity upon the Hellinger-transformed taxonomic and functional abundance matrices (see *Taxonomical and functional profiling*) and the Jaccard index upon the binary matrices (see *Metabolic network reconstruction and modeling*). Unsupervised clustering was performed upon the matrix of metabolites with $\texttt{stats\_ellipse}$ (type = “t”, level = 0.95). Hierarchical clustering was performed  upon the collapsed data, i.e., the matrix of metabolite groups, using the Jaccard index and the ward.D2 linking method, and plotted with the ComplexHeatmap v2.18.0 package [65]. For environmental data, boxplots and a principal component analysis (PCA) of soil physicochemical measurements (pH, electrical conductivity (EC), percent organic matter (OM), and fourteen others in mg/kg: N, NH_4_, NO_3_, P, K, Mg, Ca, Cu, Fe, Zn, Mn, B, S and Na) were performed with $\texttt{geom\_boxplot}$ and stats::prcomp, respectively.  The PCA was plotted with fviz_pca_biplot from the factoextra package v1.0.7. 

Elastic nets were fitted to the dataset of metabolite groups with an alpha value of 0.85 using the glmnet package v4.18 [67]. The physicochemical measurements obtained in situ were individually targeted as explained variables. Metabolite groups with nonzero coefficients and, thus, fitted as relevant for the regression model, were selected to define *key* *metabolites* if absolute values of coefficients were greater than 0.3 (Table S10). Hierarchical clustering of this processed data was performed using the Bray–Curtis dissimilarity and the ward.D2 linking method, and plotted as described above. For a visual summary of our bioinformatic pipeline, refer to Fig. 2. Lists of key metabolites were extracted and visualized with Ontosunburst v1.0.0 (https://github.com/AuReMe/Ontosunburst).

All ordination and regression analyses were performed using R v4.3.2 [66]. Plots were generated using ggplot v3.5.0 [68] unless otherwise indicated.

### Selection of minimal communities

Metage2Metabo [58] permits to select minimal communities, i.e., a set of metabolic networks of minimal cardinality that sustain the reachability of a set of targeted compounds, through the enumeration of all possible solutions.  Minimal communities are constituted by *key species* that can be either essential or alternative syntrophic organisms (referred as symbionts in the tool’s documentation). Essential syntrophic organisms appear in every solution, highlighting possible producibility bottlenecks in the microbiome, whereas alternative syntrophic organisms appear in at least one but not all solutions, suggesting functional redundancy for the targeted functions within the original dataset. We used this approach to identify MAGs putatively involved in the biosynthesis of key metabolites (see *Ordination and regression analyses*). Metage2Metabo’s $\texttt{mincom}$ command was run for each of the six sites providing a single list of all such metabolites as targets. Runs were performed with basal medium because selected metabolic groups were unambiguous across seeds, i.e., they displayed the same producibility profiles across simulations regardless of the seed set used. Minimal communities were visualized throughout power graph compression with the command m2m_analysis::workflow and styled with Inkscape v1.3.2 to reflect the taxonomy and abundance of MAGs.

## Results

### Environmentally unique sites reflect distinct taxonomic and functional profiles along the TLT

**Fig. 1:**
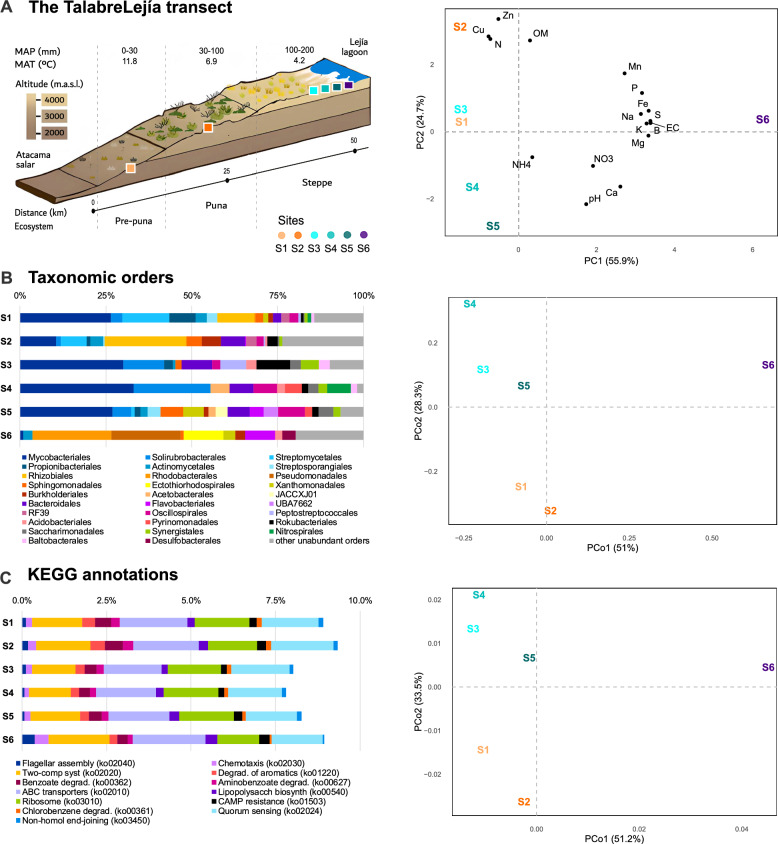
Environmental heterogeneity across the TLT. **a** Geographic illustration of the TLT adapted from Mandakovic et al. [36] showing altitude (m.a.s.l.), mean annual precipitations (MAP), mean annual temperature (MAT), and distance between sampled sites (left). Principal component analysis (PCA) conducted upon the scaled environmental data across sites (right). OM: organic matter, EC: electric conductivity. **b** Relative abundance of taxonomic orders (left). Taxa with abundance < 3% in all sites were merged into ‘other unabundant orders’. **c** Relative abundance of most dissimilar functional categories between sites following the KEGG pathway annotations (left). Ranked functions contributing up to 10% of the cumulative overall dissimilarity according to a Similarity percentage analysis (SIMPER) are shown. Importance decreases from left to right. **b–c** (right). Principal coordinates analysis (PCoA) conducted upon the Hellinger-transformed abundances for taxonomic orders (n = 72) and KEGG functional categories (n = 491). For projections for phyla, classes, and families, refer to Fig. S2, and for PFAM, COG, and CAZyme, refer to Fig. S3

Soil samples of our study span prepuna, puna, and steppe ecosystems from the western slopes of the Andes Mountains in Chile (Fig. 1a and Fig. S0). To understand how prokaryotic communities are taxonomically and functionally organized with regards to environmental changes along the TLT, 17 soil parameters were measured in situ. The geography and physicochemical measurements of the six studied sites, recently described in Andreani-Gerard et al. [37],  evidenced overall dissimilarity (Fig. 1a and Table S1). The first discriminative principal component is driven by both macro (P, S, K, Mg, and Ca) and micro (Mn, Na, Fe, and B) nutrients, which separate S6 from the rest. The second principal component orders the other five sites according to ammonium (NH_4_), environmental nitrogen (N), organic matter (OM), and two heavy metals (Cu and Zn). A clear, spread projection of these sites was observed when omitting the sample from the Lejía lagoon (Fig. S1 and Suppl. Text S1).

Alpha-diversity analyses included 146 million reads with assigned taxa (45% of the total), which were classified in 335 described prokaryotic OTUs (330 bacterial and 5 archaeal defined at 96.5% marker genes identity, Table S3). Increased richness was observed in S1 and S2, with 120 and 152 OTUs, Shannon’s indexes of 4.6 and 4.8, and Fisher’s log-series of 7.9 and 10.1, respectively (avg. S3 to S6: 40±10.6, 3.5±0.27, and 2.4±0.69). These results point to the prepuna and puna soil microbes as markedly more diverse than the sampled steppe communities.

While all sites were dominated by Actinobacteriota and Proteobacteria, as reported in previous surveys of desert soils [69–71], taxonomic profiling revealed varying microbial composition across the TLT, even at the rank of phyla (Suppl. Text S2 and Fig. S2). Examination at the rank of orders revealed little overlap of taxa between the surveyed ecosystems (Fig. 1B, Table S4), as confirmed by beta-diversity analyses separating the prepuna and puna from the steppe  along the second coordinate of the PCoA. The first coordinate strongly discriminates the Lejía lagoon’s community (Fig. 1b and Fig. S2).

To assess the impact of the observed taxonomic divergence on biological functions, we analyzed gene annotations from the PFAM, COG, KEGG pathways, and CAZyme databases (Fig. 1c and Fig. S3). A total of 8605, 4491, 429, and 125 entries from the respective databases were identified, averaging 6876 (80%), 4070 (91%), 413 (96%), and 101 (81%) different functional categories per sample (Table S5). Functional overlap was apparent at the gene level. For instance, we found 7100 PFAM annotations (83%) and 3993 COG categories (88%) that are shared in four or more sites, of which 5478 (64%) and 3652 (81%) are ubiquitous. Yet, the fraction accounting for more specific functions, particularly those found in a single site (7% of PFAM and 5% of COG), distinguish the six metagenomes in the ordination analyses (Fig. S3).

Dissimilar functional categories were ranked using pairwise comparisons of Hellinger-transformed gene abundances through SIMPER analyses (see Methods). Functional profiles are detailed in Suppl. Text S3. Briefly, S1 (prepuna) and S2 (puna) were found to be enriched in genes associated to degradation of aromatic compounds like benzoate, while S6 (lagoon) was characterized by functions associated to motility. Sites S3, S4, and S5 (steppe) showed no enrichment of the top KEGG functions contributing up to 10% of cumulative dissimilarity (Fig. 1c). PFAM functions indicate that these microbiomes are enriched in several categories related to mobile genetic elements (e.g., transposases and phage integrases) and DNA repair (Fig. S3A). We observed that the most dissimilar COG annotation was an extracytoplasmatic receptor characterizing S2, related to the uptake of tricarboxylates (TctC, Fig. S3B). Carbon metabolism was surveyed through the annotation of carbohydrate-active enzymes (CAZymes) which highlighted enrichment of glycosyl hydrolases (GH29, GH95, and GH3) in S6 and of a broad glycosyl transferase (GT4) in S1 to S5 (Fig. S3C).

Overall, functional annotations, taxonomic profiles, and physicochemical parameters, all confirm the heterogeneity of the TLT microbiomes, separating the puna and prepuna from the steppe samples and the lagoon (see Suppl. Text S4). These observations motivated a deeper exploration of the metabolism through dedicated models to generate mechanistic hypotheses on the transect’s diversity.

### A systems biology framework to simulate the environmentally-driven metabolic potential of microbiomes

Given the relationships observed between the taxonomic and functional profiles  with the physicochemical measurements, we simulated the *metabolic potential* of the TLT communities as a proxy of how they are prepared to respond to environmental shifts. In the pursuit of an ecosystem-level comprehension of microbial interactions, our framework employs metabolic networks as the basis of dynamical systems for simulation. For this, a gene catalog was reconstructed for each metagenome while 120 MAGs, accounting in average for 15.1%±4.7% of relative abundance, were obtained across sites (Table S6). Of these, 46 MAGs were classified as high-quality (avg. completeness: 94%, avg. contamination: 2%) and 74 MAGs as medium-quality (avg. completeness: 81%, avg. contamination: 2%) [75]. Metabolic networks yielded, in average, 5828±234.3 and 3774±583.5 unique reactions per sample from the assembled metagenomes and MAGs, respectively, of which more than 80% were gene-related (Table S7).

**Fig. 2:**
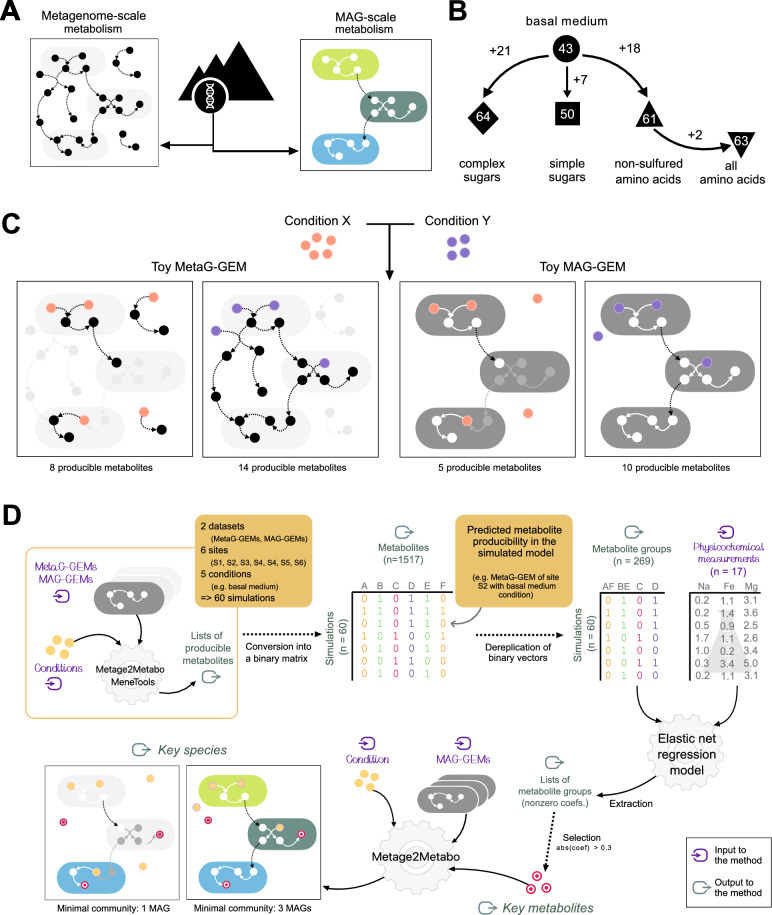
Overview of the computational framework. **a** Reconstruction of metabolic networks from sequence data. Nodes are metabolites and arrows are reactions. Each sample’s metabolism can be abstracted as a MetaG-GEM built from the community-wide metabolic network (gene catalogs, left), or as a MAG-GEM obtained from a collection of genome-resolved metabolic networks (MAGs, right). **b** Definition of conditions to be simulated (user-provided seed sets, Table 1 and Table S8). Numbers indicate how many seed compounds define each condition. **c** Toy examples of network expansion using a MetaG-GEM (left) or a MAG-GEM (right). Pink and purple circles denotes two different conditions, represented as different available nutrients. Black (resp. white) arrows and circles denote the producible metabolites in the MetaG-GEM (resp. MAG-GEM), by condition. **d** Summary of the bioinformatic pipeline used in this work. Sixty simulations of producible metabolites (scope) are performed with twelve systems and five conditions. Producible metabolites across simulations are summarized into a binary matrix. Binary vectors are collapsed into metabolite groups with identical producibility profiles across simulations. The latter are used as explanatory variables in the elastic net regression that aims at explaining soil physicochemistry. Metabolite groups significantly associated with environmental data (absolute coefficient $>0.3$) are set as targets for computing minimal communities in MAG-GEMs. Key species (colored organisms) are involved in biosynthesis throughout individual genomic capabilities (left, a single organism can produce the key metabolites) and putative cross-feeding interactions (right, several interacting organisms are necessary), see Table S11

The first step of the approach relies on the construction of two dynamical systems for each site, in the form of complementary metabolic models as an abstraction of two biological units, i.e., the whole community and a consortium of single organisms (Fig. 2a). On one hand, metagenome-scale models (*MetaG-GEMs*) inform about the collective metabolism of a community, independent of the taxa performing the functions. On the other, building models based on MAGs (*MAG-GEMs*) enables the study of putative populations that, although representing only a fraction of all prokaryotic species inhabiting the TLT, are abundant enough to be captured by genomic assemblies. Thus, the MAG-scale approach can be understood as a compromise that provides taxonomic identity to a subset of functions that are likely to be relevant within the corresponding communities, at the cost of possibly losing reactions from contigs that could not be binned. Overall, the objective of our strategy based on explainable models is to identify metabolic drivers associated with each site and to determine taxa associated to their synthesis, taking into account metabolic complementarity.

The second step of the method defines simulation conditions for the dynamical systems. Considering that the Atacama Desert exhibits oligotrophic conditions and, thus, organic carbon and nitrogen supplies are expected to be scarce, we provide five scenarios simulating different nutritional sources (Fig. 2b). A *basal medium* contains a limited set of coenzymes, metal ions, and inorganic compounds with CO_2_ and HCO_3_ as carbon sources. Two nutritional conditions explore the effect of organic carbon, specifically, by adding carbohydrates that enter glycolysis directly (*simple sugars*) or others, more recalcitrant, commonly found in soils (*complex sugars*). Finally, to assess the effect of supplying organic nitrogen, we defined two conditions with amino acids (*all amino acids* and *non-sulfured amino acids,* see Methods, Table 1 and Table S8).

The third step consists in running the simulations and computing the *metabolic potential*, i.e., the response of the dynamical system to the simulated conditions, described as sets of metabolites predicted to be producible. To provide an overview of the metabolic functions of microbiomes, our formalism uses a concentration-independent Boolean semantic. We illustrate in Fig. 2c the impact of two initial conditions on the producibility of metabolites in a toy MetaG-GEM and its corresponding toy MAG-GEM. We observe that functions present in the metagenome but absent from MAGs may alter the number and identity of producible metabolites that depend on the simulated conditions. In total, using the data from the TLT, 60 simulations were conducted, accounting for the five conditions and the two systems for each of the six sites (Fig. 2d).

The fourth step of the  strategy involves regression analyses and targeted simulations (Fig. 2d). Briefly, producible metabolites were collapsed into *metabolite groups* if producibility profiles across simulations were identical, and then associated with the physicochemical measurements of sites. The strongest associations are used to select *key metabolites*, set as input in downstream simulations when predicting minimal communities of MAGs involved in their biosynthesis. We detail in the following sections the outcomes of this systems biology framework.

### Microbial metabolism outlines the adaptative potential of communities in demanding environments

**Fig. 3:**
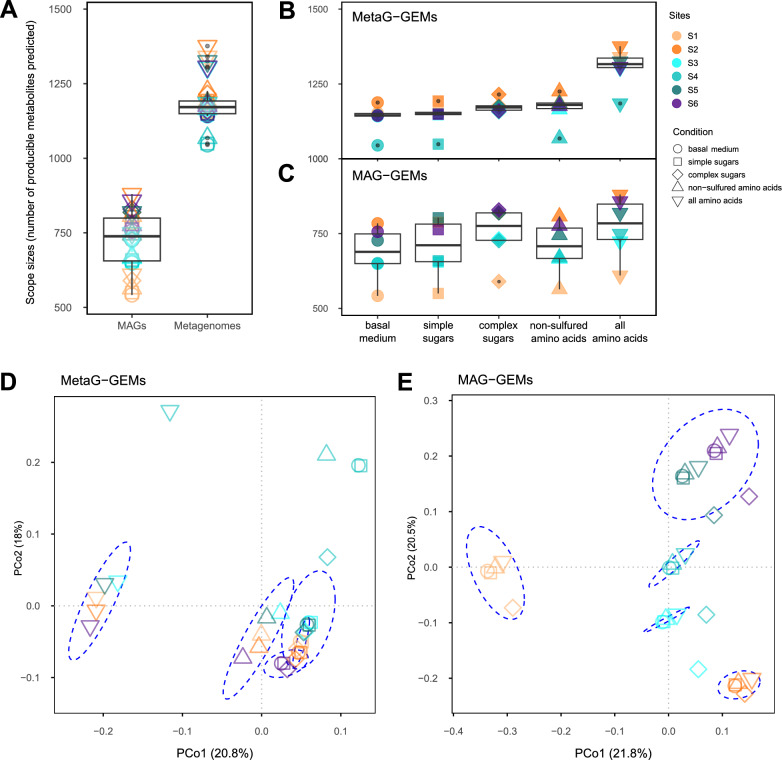
Quantitative description and ordination analysis of producible metabolites. **a** Distribution of the number of producible metabolites across all 30 simulations (6 sites, 5 conditions) in MAG-GEMs and MetaG-GEMs. **b, c** Number of producible metabolites according to the simulated conditions in MetaG-GEMs (**b**) and MAG-GEMs (**c**). **d, e** PCoA obtained from the presence/absence matrix of producible metabolites for MetaG-GEMs (**d**) and MAG-GEMs (**e**)

Results of the simulations are presented in Fig. 3 and Fig. S4. Across sites, 1517 unique metabolites were predicted to be producible in MetaG-GEMs, of which 1166 (76.9%) were captured in MAG-GEMs. Producible metabolites ranged on average from 1136.3 ± 47.9 with *basal medium* to 1306.2 ± 64.9 with *all amino acids* in MetaG-GEMs, and from 684.8 ± 88.9 to 773.3 ± 100.3 in MAG-GEMs, respectively (Fig. 3a). As expected, the MAG-GEMs—that encompass a reduced portion of the microbiome—exhibit smaller scopes than those obtained through the MetaG-GEMs, regardless of the simulated conditions. Within dataset, the number of producible metabolites depends on the simulated condition (Fig. 3b,c). The highest number of producible metabolites in MAG-GEMs is exhibited using *complex sugars* and *all amino acids*, while the largest scopes in MetaG-GEMs are retrieved with the latter. This increased metabolic potential in *all amino acids* for both MAG- and MetaG-GEMs suggests that the addition of organic sulfur, i.e., cysteine and methionine, has a strong effect compared to solely organic nitrogen, simulated with *non-sulfured amino acids*. Additionally, S2 (puna) reached the largest metabolic potential regardless of the conditions in the MetaG-GEMs, while the S1 (prepuna) and S4 (steppe) consistently exhibited the lowest number of predicted metabolites in MAG- and MetaG-GEMs, respectively. Core metabolites were defined as those found to be producible in all 30 possible combinations of sites and conditions by dataset. Core metabolites prevail in both datasets, comprising 61.2% and 40.5% of all compounds in MetaG-GEMs (n = 928) and MAG-GEMs (n = 472), respectively (Table S9).

An ordination analysis was performed to compare producible metabolites across sites and conditions (Fig. 3d, e). Results show that MetaG- and MAG-GEMs exhibit different metabolic profiles. On one hand, the high proportion of core metabolites predicted from the MetaG-GEMs determines the overlap of all sites but S4 (see Suppl. Text S3) when carbon is provided with *basal medium*, *simple sugars*, and *complex sugars*. While profiles of producible metabolites are more dissimilar in the *all* and *non-sulfured amino acids* conditions, the same five sites still cluster together. This underlines the strong impact of organic sulfur when compared to organic nitrogen and organic carbon, already implied by Fig. 3b. The effect of conditions on the simulated metabolic profiles, rather than the effect of sites themselves, suggests high functional redundancy across microbiomes (Fig. 3d). On the other hand, most metabolic profiles of MAG-GEMs cluster by site (Fig. 3e) indicating that the subset of organisms from each ecosystem harbor different metabolic pathways, specifically fitted to the physicochemical properties and nutrient contents along the TLT. These results suggest that microbial communities as a whole could act as a reservoir of functions with little variability across sites (Fig. 3d), whereas the most abundant players in each community, retrieved as MAGs, exhibit larger differences in their metabolic responses across sites (Fig. 3e).

### Key metabolites reveal carbon and nitrogen pathways driven by the puna and lagoon environments

The binary matrix of producible metabolites (n = 1517, Fig. 2d) was dereplicated by collapsing metabolites with identical producibility profiles across all 60 simulations into 269 *metabolite groups*. Among those, 45% (n = 121) harbored a single metabolite. The median and average size of a metabolite group were 2 and 5.6, respectively. The largest group, the one intersecting core metabolites from both datasets, contained 469 metabolites. We assessed those core metabolites throughout their structural ontology according to MetaCyc and found that they span a wide variety of chemical compounds including carboxylates (n = 98, 21%), nucleosides (n = 77, 16%), glycans (n = 72, 15%), heterocyclic-organonitrogen (n = 69, 15%), amino acids (n = 46, 10%), and ions (n = 27, 6%). For further details, refer to the corresponding supplementary HTML file. Hierarchical clustering of producibility vectors across simulations (Fig. S5) revealed two main clusters: 211 metabolite groups that largely varied across sites but were rather insensitive to conditions (Fig. S5A), and 58 metabolite groups mostly influenced by amino acid-containing conditions while overall consistent across sites (Fig. S5B). In the latter cluster 16 metabolite groups are associated to *non-sulfured amino acids* and 42 to the inclusion of cysteine and methionine. Biochemical families that distinguish sites across simulations are described by dataset in supplementary HTML files. Results highlight that  missing functions in the MetaG-GEM from S4 are related to biosynthesis of polysaccharides, glycoconjugates, terpenoids, and of various lipids. In MAG-GEMs, those functions were only present for the puna (S2).

**Fig. 4:**
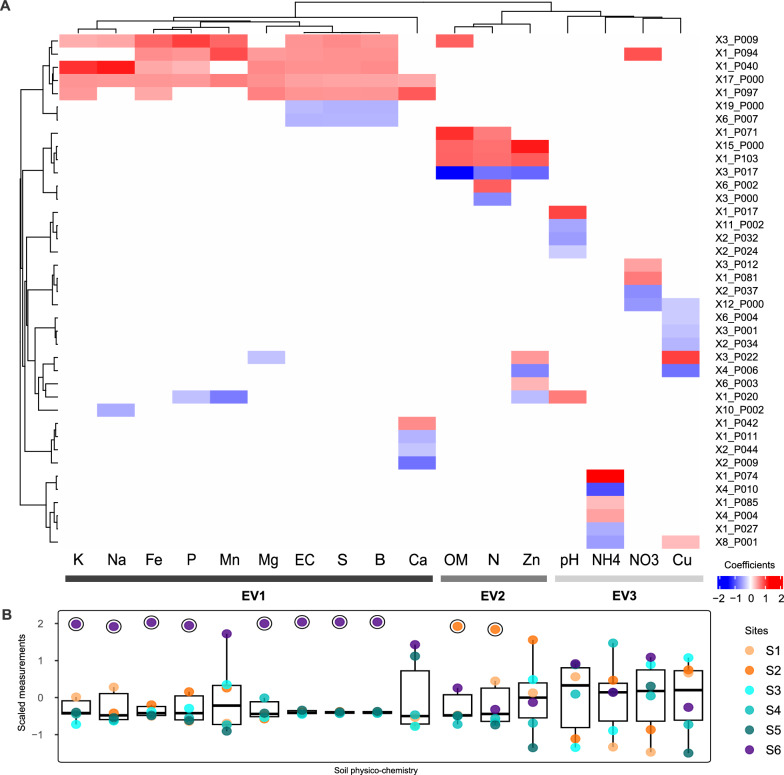
Metabolite groups proposed to be driven by the environmental pressures of the TLT. **a** Heatmap of metabolite groups selected with an elastic net regression model as the best predictors for explaining environmental data. The first term of row labels indicates the number of metabolites in each group. Data is shown if the absolute value of the fitted coefficient was $\ge$0.3 (for the unfiltered data, see Table S10). A total of 171 key metabolites emerged from these 39 metabolite groups (rows). Hierarchical clustering of columns enabled three groups of environmental variables (EVs) to arise. **b** Boxplots show the scaled values of in situ soil physicochemical measurements comprising the environmental data. Outliers are displayed inside black circles. OM: organic matter, EC: electric conductivity

We used a regression model to associate the in situ physicochemical measurements (n = 17, Fig. 1a) with the predicted metabolite groups (Fig. 2d). Our results show that 82 out of the 269 unique metabolite groups (30.1%) had nonzero coefficients, meaning that 292 out of the 1517 unique metabolites (19.3%) were fitted as relevant for at least one environmental variable (Table S10). Since coefficient values account for the strength and direction of the corresponding relationship, we focused on metabolite groups with absolute values greater than 0.3 (n = 39), comprising 171 elements which we refer to as *key metabolites*. A hierarchical clustering of the selected metabolite groups revealed three main clusters of environmental variables (EV1, EV2, and EV3, Fig. 4a). Namely, EV1 is defined by K, Na, Fe, P, Mn, Mg, S, B, Ca, and electric conductivity; EV2 by organic matter , Zn, and N; and EV3 by pH, Cu, NO_3_, and NH_4_. We observed that EV1 and EV2 mainly cluster physicochemical parameters for which soils from the Lejía lagoon (S6) and the puna (S2) are outliers, respectively (Fig. 4b). This implies that such parameters likely constitute major abiotic stressors for microorganisms inhabiting these rare ecosystems, whereas EV3 has no evident relation to geography.

We further surveyed the link between sites and metabolite groups associated with the first two clusters of environmental variables. Firstly, EV1 was positively associated to five metabolite groups representing 23 key metabolites linked to nitrogenated pathways (e.g., L-leucine, L-valine, L-arginine, N-acetylneuraminate, and N-acetylmannosamine degradation, L-isoleucine biosynthesis) and osmotic stress (see Suppl. Text S5). Secondly, EV2 was positively associated to two metabolite groups representing 23 key metabolites mostly linked to carbon cycling (e.g., staurosporine, violacein, and flavonoid biosynthesis, and aromatic degradation). This observation is consistent with, and further expands the results obtained via functional profiling, showing enrichment in some functions related to carbon catabolism and transport in the puna (Fig. 1c, see Suppl. Text S6). For structural ontology of key metabolites from the puna (EV2) and the lagoon (EV1) explore the interactive HTML files provided in supplementary_file3.zip.

### Minimal communities of key species display distinct structural properties along the TLT

**Fig. 5:**
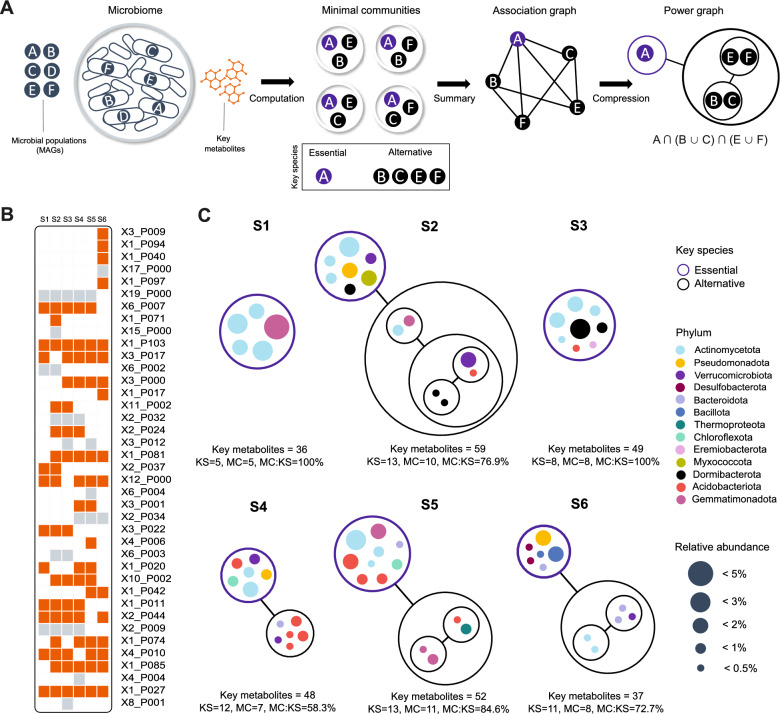
Minimal communities potentially able to produce key metabolites. **a** Illustration of the reverse engineering simulation protocol for identification of MAGs involved in the biosynthesis of key metabolites with Metage2Metabo [58], see also Fig. 2d. **b** Panel showing the producibility of selected metabolite groups (n = 39) containing key metabolites (n = 171) by site (see Table S11). Orange squares depict metabolite groups (n = 27) containing key metabolites (n = 81) that are producible in MAG-GEMs. Gray squares depict metabolite groups (n = 12) containing key metabolites (n = 90) that were only producible in MetaG-GEMs and, thus, had no effect in the computation of minimal communities, which can only be carried out with the genome-resolved approach. The first term of row labels indicate the number of key metabolites in each metabolite group. **c** Power graphs summarizing the structure of all predicted minimal communities per site. Blue and black circles should be interpreted as “AND” and “OR”, respectively, meaning that all MAGs inside blue circles are required for the production of the key metabolites provided as targets (essential syntrophic organisms) whereas only one MAG is needed inside each black circle (alternative syntrophic organisms). Lines represent the sequence of decision-making for the combinatorics of possible solutions and should be interpreted as “AND”. Relative abundance represents the percentage of metagenomic reads mapping to each MAG, i.e., microbial populations in panel (A). KS: key species, MC: minimal community

To identify the *key species * [58] involved in the producibility of at least one of the 171 key metabolites, we set up new simulations using the MAG-GEM of each site and basal medium, and computed minimal communities taking into account metabolic complementarity (Figs. 2d and 5a, see Methods). This reverse engineering approach  captures a refined signal connecting the environment with key species throughout key metabolites.

The first observation was that 90 key metabolites were produced only in MetaG-GEMs simulations (Fig. 5b). Although precise information about the taxa responsible for their production cannot be inferred, these 90 metabolites constitute the environmentally-driven signal for whole communities. Hence, despite the MetaG-GEMs demonstrating a high functional overlap, as pointed out earlier, this method flags metabolic functions specifically related to geography and soil physicochemistry (Fig. 3d). We therefore assessed the producibility of the remaining 81 key metabolites (27 metabolite groups) in the six sites (Fig. 5c). Our findings show that, out of the 120 MAGs, 62 are predicted to be key species. From these, 41 MAGs were *essential syntrophic organisms* (Fig. 5c), meaning that they occurr in all predicted minimal communities and, thus, constitute putative load points for the network (Tables S6 and S11). This suggests that one third of the reconstructed MAGs carry unique metabolic machineries related to the biosynthesis of at least one key metabolite. In average by site, MAGs can produce 46.8±8.9 key metabolites, of which 26.3±6.1 were predicted to be producible only through cooperative mechanisms (Table S11).

We dived further into the structure of communities per site by enumerating all possible minimal communities involved in key metabolite biosynthesis and analyzing the co-occurrence patterns of selected MAGs and their combinations (see Methods). Key species denote the functional redundancy of the microbiome with respect to the production of targeted metabolites when their number is larger than the size of the minimal community. In other words, some solutions may predict one key species over another without altering the ability for the minimal community to produce the targets. Here,  the predicted minimal communities exhibit different microbial structures regarding the number and type of  key species they comprise (Fig. 5c). Some sites (S1, S3) led to rigid communities: a unique consortium is able to sustain the producibility of key metabolites, meaning that no MAG could be removed or replaced while preserving the community’s biosynthetic potential (MC:KS=100%, MC: minimal community, KS: key species). These theoretical abstractions indicate more fragile systems, where environmental perturbations could directly compromise the ability of the community to elaborate a metabolic response [76]. On the opposite side, we found more complex structures that range from many replacements for a single function like in S4 (MC:KS=58.3%), to a couple of replacements for a few functions like in S2, S5, and S6 (MC:KS>70%). These *alternative syntrophic organisms* increase the combinatorics of possible community assemblies a soil microbiome can resort to in order to reach the targeted metabolic products. In these cases, metabolic redundancy suggests robustnes to changes in community composition upon environmental circumstances [11]. Such reserve of functions offers plasticity and, ultimately, resilience to the whole communities [77].

We then surveyed the abundance of these MAGs regarding metagenomic reads per sample. On average, key species accounted for 8.8% of total relative abundance per site. Site S6 diverged since key species there accounted only for 3.5% of the total abundance, which suggests that microbes sampled from the Lejía lagoon probably have a complex underlying structure that our approach overlooked because of assembly limitations. Nevertheless, this result also implies that unabundant prokaryotes are likely to catalyze critical metabolic steps in ecosystem functioning, as argued by Wang et al. [78]. On the other hand, we observed that MAGs from S1, all defined as essential syntrophic organisms, encompassed the highest cumulative abundance of key species (13.1%) across the TLT ranging from 1.6% to 4.7% per MAG, compared to an average of 0.8% for essential syntrophic organisms from other sites. This observation suggests that the metabolic response of  some TLT communities may fall on a few abundant members, likely due to the successful strategies they employ in demanding environmental conditions that severely limit other microbes to thrive [79]. Regarding taxonomy, we observed key species of Actinomycetota and Acidobacterota in every site, suggesting that members from these phyla play critical roles in community structure and stability, especially Actinomycetota that occur in every proposed consortium (Fig. 5c). For a detailed taxonomic description of minimal communities, refer to Suppl. Text S7.

Overall, by defining environmentally-driven metabolites as functions of interest, our strategy is able to classify key species as essential or alternative syntrophic organisms, combine this information with taxonomic and abundance data, and describe microbial communities as more resilient or more sensitive to environmental shifts. This targeted approach identifies critical roles and players in microbiome functioning and provides a customizable pipeline for studying microbial metabolism in natural environments.

## Discussion

### A functional description of the TLT using metabolic modeling

In this work, we characterize the metabolic potential of six contrasting microbiomes from an altitudinal gradient in the Atacama Desert, and explore their relationship with soil physicochemical data by using a systems biology strategy. In general, our results show that the addition of organic carbon in the forms of simple and complex sugars do not signify a major change in the predicted scopes of the TLT microbiomes when compared to simulations upon inorganic carbon sources only. Although carbon fixing members are known to be scarce in soils [80], our abundance-independent approach underscores the ability of those few members that contribute to carbon uptake for the whole community. We also observed an increased sensitivity towards the input of organic sulfur, in contrast to organic nitrogen, that highlights the relevance of methionine and cysteine as precursors in metabolite biosynthesis. Methionine and cysteine are the only sulfur-containing amino acids incorporated into proteins. Methionine, in the form of N-formylmethionine, initiates the synthesis of nearly all prokaryotic proteins, while S-adenosylmethionine, a highly versatile cofactor, is involved in methyl and 5’-deoxyadenosyl group transfers, polyamine synthesis, and numerous other processes. In contrast, cysteine forms disulfide bonds that determine protein structure and participate in protein-folding pathways [81].

Given that key metabolites comprised a small fraction of the simulated scopes, we were able to sharpen the differences between sites, retrieved via metabolic modeling, and further link them with seventeen environmental drivers. On one side, the capabilities for pigment and antioxidant biosynthesis and for aromatic degradation of the puna microbiome were related to the high contents of organic matter, nitrogen, and zinc in that area. A study assessing the functional potential of soil microbes associated to transitional hygrophilous plants from the Atacama Salar showed an enrichment of certain metabolic pathways for the degradation of organic matter and aromatic compounds and the biosynthesis of amino groups [82]. We propose that the distinctive vegetation belt that characterizes the puna ecosystem [83] is a critical source of nitrogen throughout the exudation of organic compounds into the soil that also chelate zinc, immobilizing it for slow release. This would significantly improve carbon and nitrogen availability in an ecosystem adapted to extreme nutrient limitations [84].

On the other side, several alternative pathways for amino acid biosynthesis and degradation detected at the Lejía lagoon’s shore were driven by abiotic factors related to salinity, electric conductivity, and sulfur, among others. The chemical structures of some of these nitrogenated compounds classified them as short chain fatty acids and glycans, suggesting that they could contribute to the metabolic response to temporal shifts in water availability by improving water retention and serving as energy reservoir [85, 86]. These results complement our recent work assessing specialized metabolism of natural products on the same soil sampless that highlighted some bacterial members of the lagoon microbiome with niche-adaptations for the acquisition of organic nitrogen and for osmotic stress resistance throughout heterocyst glycolipid-like mechanisms and ectoines [37].

### MAGs highlight emergent properties of functionally redundant gene reservoirs

As expected from the redundant functional profiles, the metabolic potential recovered from the TLT considerably overlapped across the sampled ecosystems, where more than 60% of producible metabolites predicted with the community-wide approach were found in every site, regardless of the simulated condition. This is consistent with previous observations made on 845 soil communities across 17 climate zones around the globe where, due to functional redundancy, microbial functions based on gene abundances were more stable regarding geography than taxonomy and soil properties [87]. Another effort surveying 962 metagenomic studies from nine different ecosystems showed that, while metabolic overlap in soils is overall lower compared to other environments (e.g., marine and freshwater), extreme environments were the most functionally redundant [88]. Given that functional redundancy is a necessary condition for taxonomic turnover within functional groups [89], we hypothesize that the microbial communities inhabiting the TLT depend on a fairly exhaustive *gene reservoir* to withstand the environmental perturbations they may sporadically encounter in such extreme conditions. Nevertheless, our regression-based strategy distinguished 90 compounds that account for the metabolic differences between sites, at first sight hidden behind the redundant communities.

In this sense, it has been speculated that populations with similar metabolic repertoires may specialize on distinct nutrients and, thus, express separate ‘realized’ niches—rather than ‘fundamental’ niches—at the transcriptional level [89, 90]. Considering that the metabolic functions are finely tuned by the environment including the presence and activity of other community members, only a few populations of a same functional group may emerge to actively perform a given function. Thus, while some organisms exhibit alternative modes to gain energy, others may simply be inactive due to differing enzyme efficiencies, growth kinetics, and other traits influencing their growth rates under specific conditions [89]. The latter would explain why MAGs in our dataset display a site-driven metabolic behavior fitted to their corresponding geographies, with little functional overlaps. Therefore, we propose that the genome-resolved approach offers valuable insights into the emergent properties of metabolisms operating in specific contexts. Reconstructing MAG-scale metabolic networks is particularly useful for identifying adaptations that enable microbes to thrive in natural environments.

Assessment of MAGs enabled us to identify key species involved in the biosynthesis of key metabolites, this is, showing an association with the physicochemical environment. These key species are classified as essential or alternative syntrophic organisms depending on whether they are strictly required for the production of the target metabolites  or they can be functionally replaced [58]. Although it is uncertain that MAGs herein defined as essential exert a critical role in organizing the structure of their respective soil microbiomes, they exhibit unique metabolic functions that connect tightly with biosynthetic steps found in the genomes of other community members [3, 9] and, hence, might constitute keystone taxa [91]. We hypothesize that some of these metabolic dependencies prompt putative cooperation events through cross-feeding and that the removal of the proposed keystone taxa would alter microbiome stability, potentially causing downstream impacts on ecosystem processes [5]. Lastly, we acknowledge that our approach may have overlooked other organisms displaying keystone roles due to limitations in the genomic assemblies related to low abundance [29] and that, even though minimal communities is a reasonable mathematical solution for delving into ecological functioning of microbiomes, it may not comprehensively reflect the (non-minimal) mechanisms employed in nature.

### Advantages and limitations of metabolic models for deciphering microbiome functioning

Our systems biology framework, a methodological contribution of this work, relies on dynamical modeling for elucidating the metabolic potential of whole communities and consortia of single organisms  from complex ecosystems like desert soils. The use of constrained-based models such as flux balance analysis (reviewed in Cerk et al. [92]) would hardly be applicable in a context where metabolic networks were reconstructed automatically and curation is limited for non-model organisms. The use of ordinary differential equations, on the other hand, would require setting up many parameters like kinetic constants which are largely unknown in natural environments. Here, the discrete model of metabolic producibility is an approximation of those quantitative approaches that predicts fixed points of the dynamical system and offers flexibility [24]. Implemented in a logic paradigm via an expansion algorithm [58, 93, 94], this approach enables to go beyond pathway description and assess the impact of environmental factors on metabolism as well as its sensitivity to nutrients from different organic sources.

The strategy generates community-wide and genome-resolved metabolic models One provides an overview  of ecosystem-level functions captured in the gene catalogs, and the other provides information on the relationship between taxonomy and functions retrieved in MAGs. Although both cases underestimate the complexity of the community, they complement each other and enhance reliability of bottom-up inferences. Regardless its utility for addressing relevant questions in microbial ecology research, our framework inherently incorporates many simplifications that warrant explicit discussion.

The study focuses on the metabolic potential, i.e., the putative metabolic capabilities of prokaryotic communities, where Boolean semantics imply that metabolic reactions, enzyme biosynthesis, and metabolite transports are cost-free. This choice necessarily overestimates what happens in situ, where such processes have a strong impact on microbial adaptation, specially, in environments with scarce nutrient availability [95–97]. From an ecological point of view, this can be understood as the theoretical space within which microbes can collectively assemble a metabolic response, denoting the upper bound of metabolic interactions. The assumption is sustained on previous knowledge stating that metabolite exchanges are frequent in microbial communities and expected to mean low cost for fitness [98]. In fact, some  cutting-edge efforts rely on the “superorganism” concept [99, 100], where all biological units in a community are interconnected beyond cell-wall barriers, recovering highly valuable information of microbial systems [27].

While assuming that any metabolite producible by a taxon is available to any other member of the community risk false positive cross-feeding predictions, this simplification stems largely from undocumented transporter annotations and is exacerbated by the scarcity of experimental data for uncultured microbes [101]. Metabolic databases remain incomplete, with a substantial fraction of microbial proteins lacking an assigned function. This knowledge gap hinders critical aspects of community metabolism, particularly for understudied lineages. The challenge is especially acute when modeling complex natural microbiomes, where unannotated enzymes, transporters, and transcription factors impede rigorous network reconstruction and curation; however, this limitation is transversal to the field. Improvements can be done at the metagenomic sequencing step by using both short- and long-reads to  enhance the quality of the resulting metabolic models [92].

All taken together, simplifying assumptions are indispensable when dealing under the paradigm of missing information. In this context, mechanistic models offer unparalleled advantages for elucidating fundamental metabolic processes and their causal relationships [102]; nonetheless, their predictive power would benefit significantly from empirical validation. For example, targeted metabolomics and microcosm experiments could synergize with our in silico approach by quantifying predicted metabolites under controlled exposure to varying conditions [83, 103]. Observational studies focusing on community shifts, keystone taxa, and metabolic resilience are needed for understanding complex microbiomes like those inhabiting desert soil ecosystems, and could benefit from predictive models like ours.

## Conclusions

We studied the metabolic potential of soil microbiomes from an altitudinal gradient in the Atacama Desert. To do so, we conceived a modeling framework, suitable for non-model microorganisms and scalable for large datasets, that facilitates progress toward disentangling the complex metabolic interactions that shape microbiome functioning. With few inputs, i.e., nucleotide sequence data, custom nutritional scenarios, and soil physicochemical measurements, we link metabolic insights with taxonomy and, more importantly, with the environment. By assessing the metabolic potential under varying conditions for infering key metabolites, environmental drivers, and key species, we  uncover niche adaptations evolved along the TLT such as the influence of soil organic matter and zinc on aromatic degradation, and of salinity and other stressful abiotic factors on nitrogen cycling. We also capture the crucial role of organic forms of sulfur in this oligotrophic environment that stood over the impact of carbon and nitrogen on microbial metabolism. Finally, our  modeling strategy  fathoms the functional overlap often found in metagenomes as a gene reservoir that provides microbial communities means to adapt to future environmental shifts. We demonstrate that MAGs representing single populations exhibit divergent properties, emergent from microbiome functioning and tailored to the current environment, and we further propose some mechanisms of cooperation they might employ to withstand them.

## Additional file

Supplementary file 1. Tables S1 to S11.

Supplementary file 2. Figures S0 to S5, Table S12 and Supplementary Texts S1 to S7.

Supplementary file 3. Interactive HTML files of core and key metabolites' structural ontology.

## Data Availability

Nucleotide sequences of metagenomes and MAGs are deposited at the NCBI database under the BioProject accession PRJNA1104199. Metabolic networks are deposited at Zenodo 10.5281/zenodo.14537000. Ordination and regression analyses and plots can be reproduced with the script deposited at https://github.com/cmandreani/models_Atacama.
